# Supplementary material for: Complex Networks Analyses of Antibiofilm Peptides: An Emerging Tool for Next-Generation Antimicrobials’ Discovery
Source: Antibiotics (Basel). 2023 Apr 13;12(4):747. doi: 10.3390/antibiotics12040747 (PMC10135022; doi:10.3390/antibiotics12040747)
Supplement: Supplementary file 1 [file antibiotics-12-00747-s001.zip › Supplementary_Materials_Final/FigureS3_Motif_Detection_by_Sequence_Alignments_per_Network_Clusters.pdf]

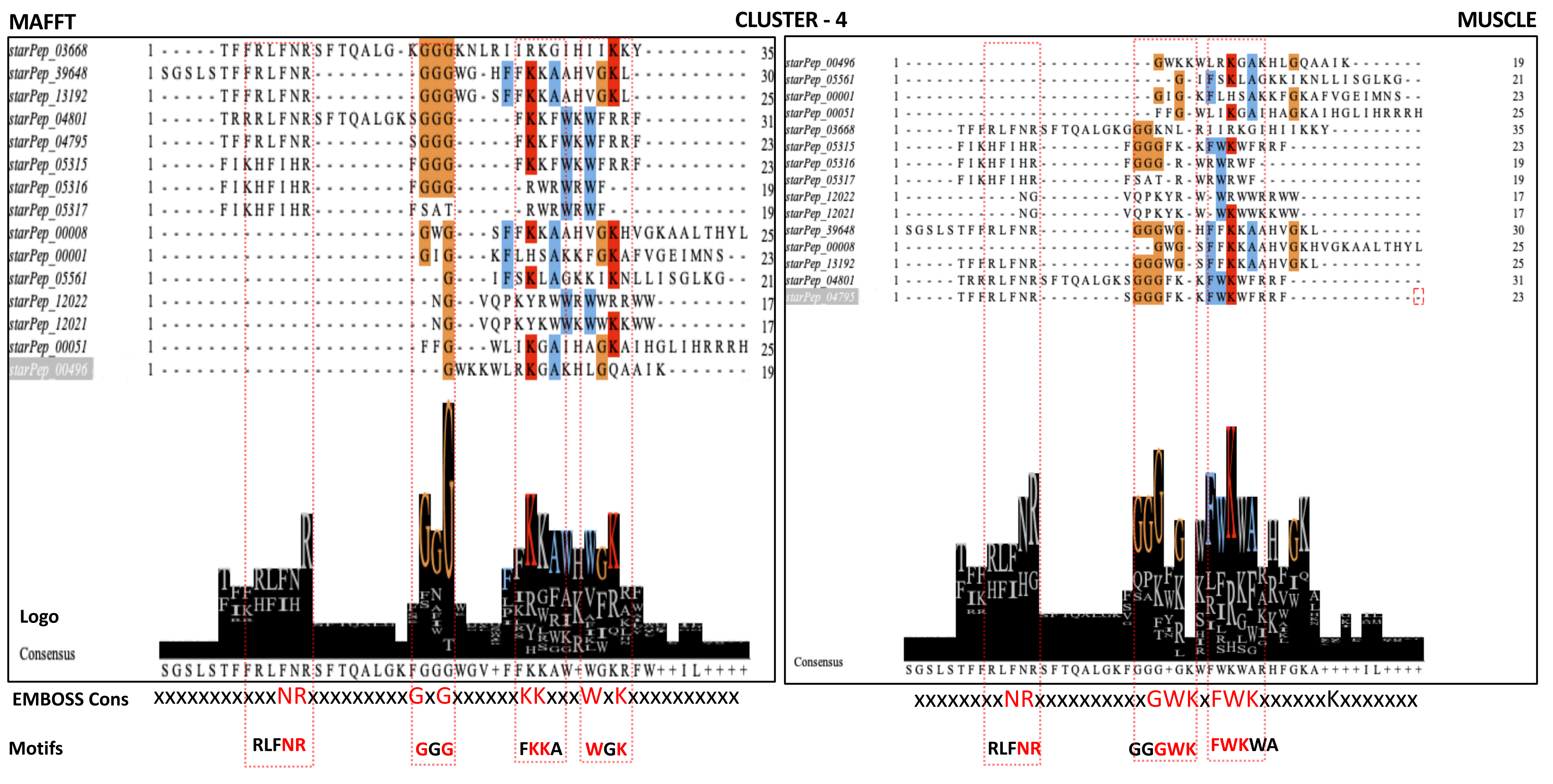
MAFFT
CLUSTER - 4
MUSCLE
Logo
Consensus
SGSLSTFFRLFNRSFTQALGKFGGGWGV+FFKKAW+WGKRFW++IL++++
SGSLSTFFRLFNRSFTQALGKFGGG+GKWFWKWARHFGKA++++IL++++
EMBOSS Cons
xxxxxxxxxxxNRxxxxxxxxxGxGxxxxxxKKxxxWxKxxxxxxxxxxx
xxxxxxxxNRxxxxxxxxxxGWKxFWKxxxxxxKxxxxxxx
Motifs
RLFNR
GGG
FKKA
WGK
RLFNR
GGGWK
FWKWA

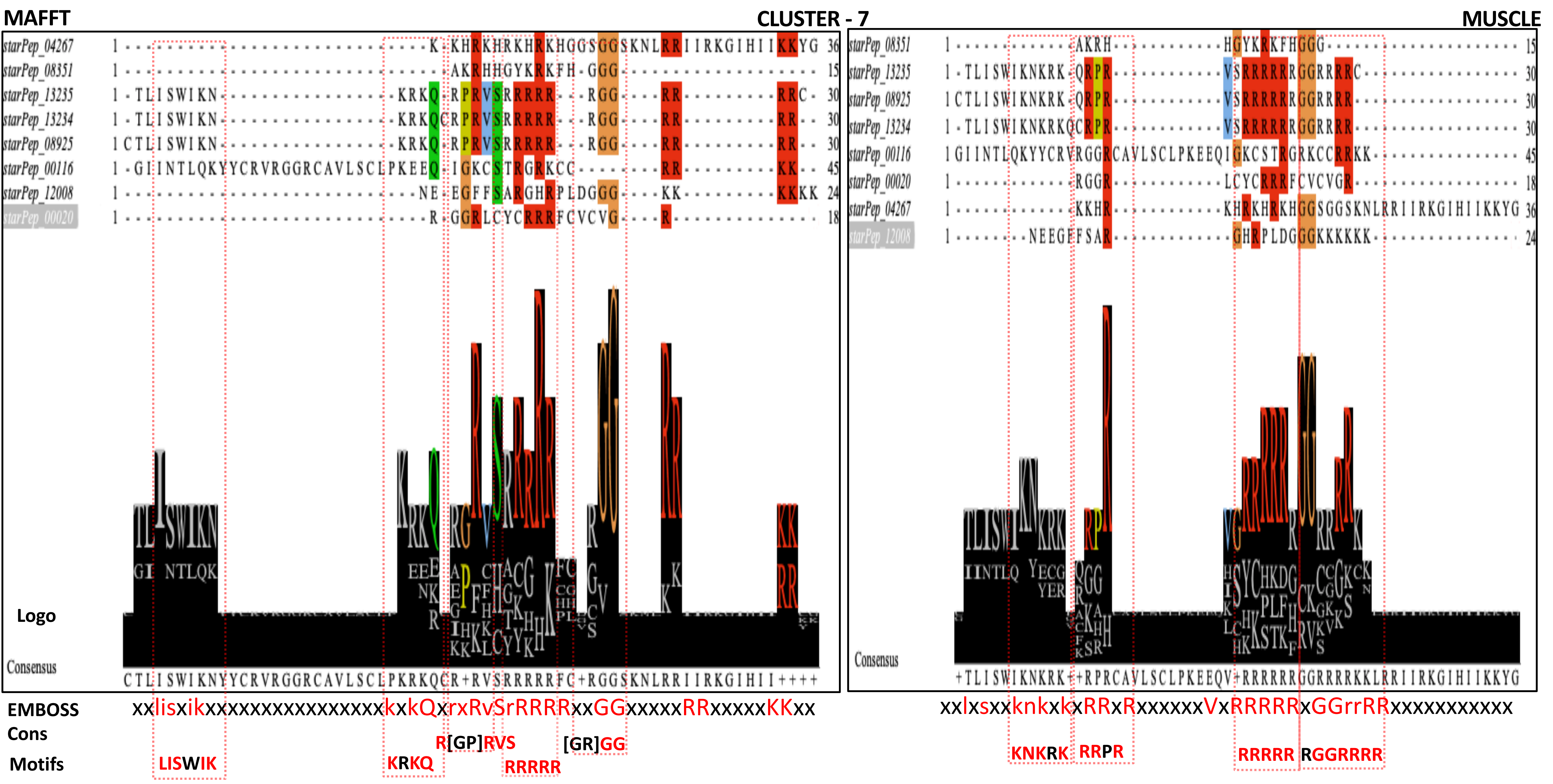
MAFFT
CLUSTER - 7
MUSCLE
Logo
Consensus
EMBOSS Cons
Motifs
LISWIK
KRKQ
R[GP]RVS
RRRRR
[GR]GG
KNKRK
RRPR
RRRRR
RGGRRRR

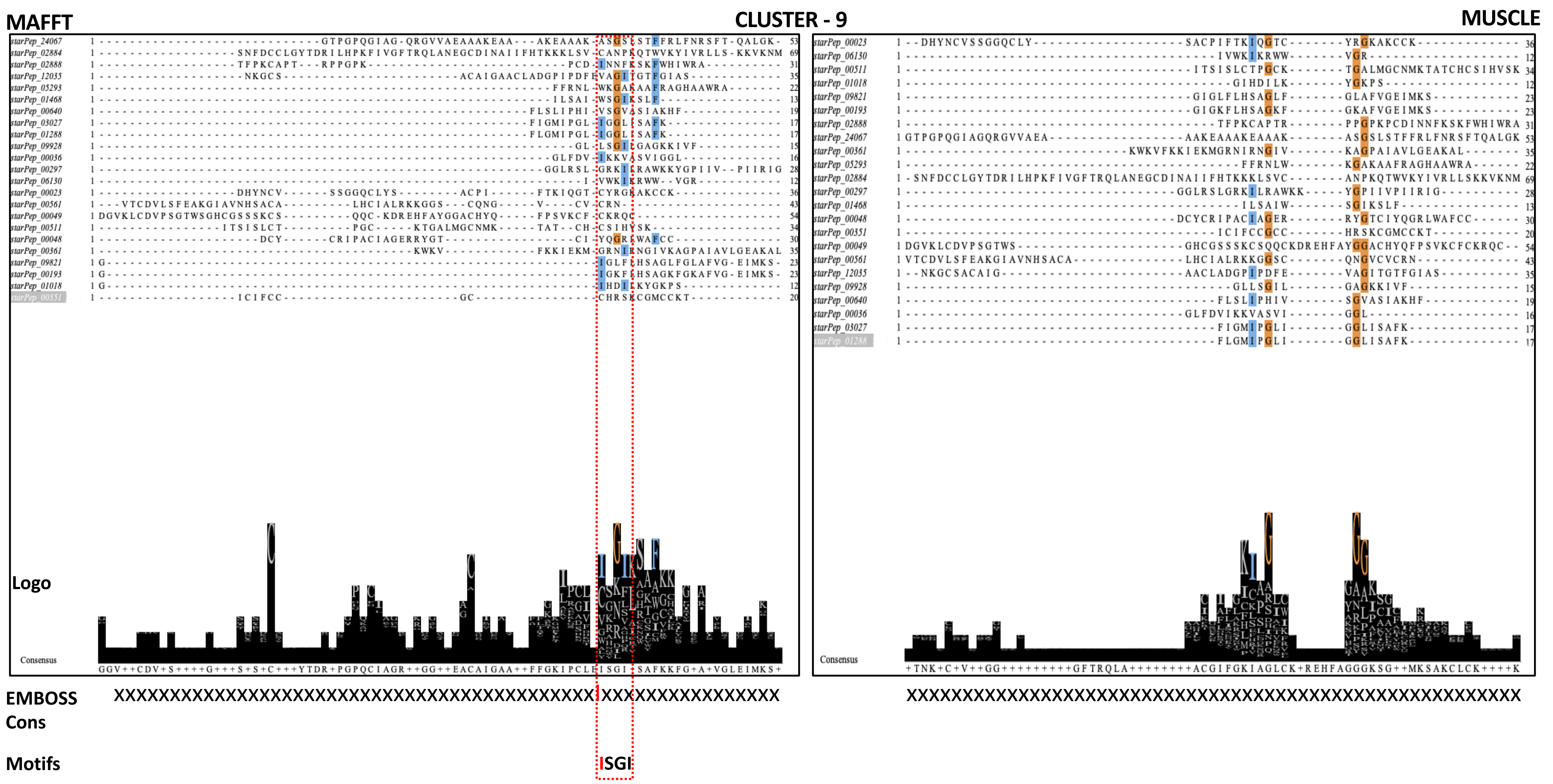
MAFFT
CLUSTER - 9
MUSCLE
Logo
Consensus
EMBOSS Cons
Motifs
ISGI

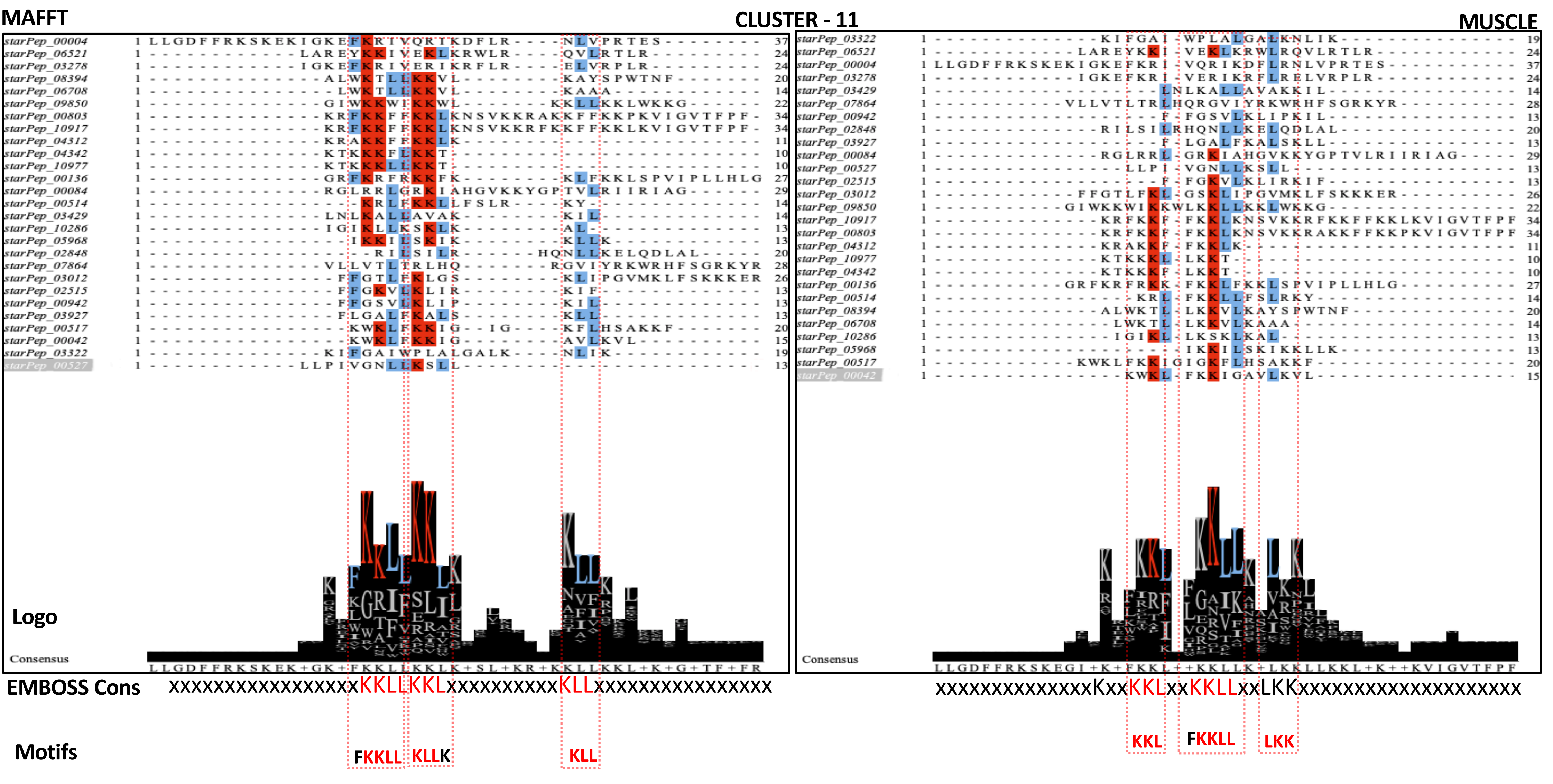
MAFFT
CLUSTER - 11
MUSCLE
Logo
Consensus
EMBOSS Cons
xxxxxxxxxxxxxxxxxKKLLKKLxxxxxxxxxxKLLxxxxxxxxxxxxxxxxx
xxxxxxxxxxxxxxKxxKKLxxKKLLxxLKKxxxxxxxxxxxxxxxxxxxxxx
Motifs
FKKLL
KLLK
KLL
KKL
FKKLL
LKK

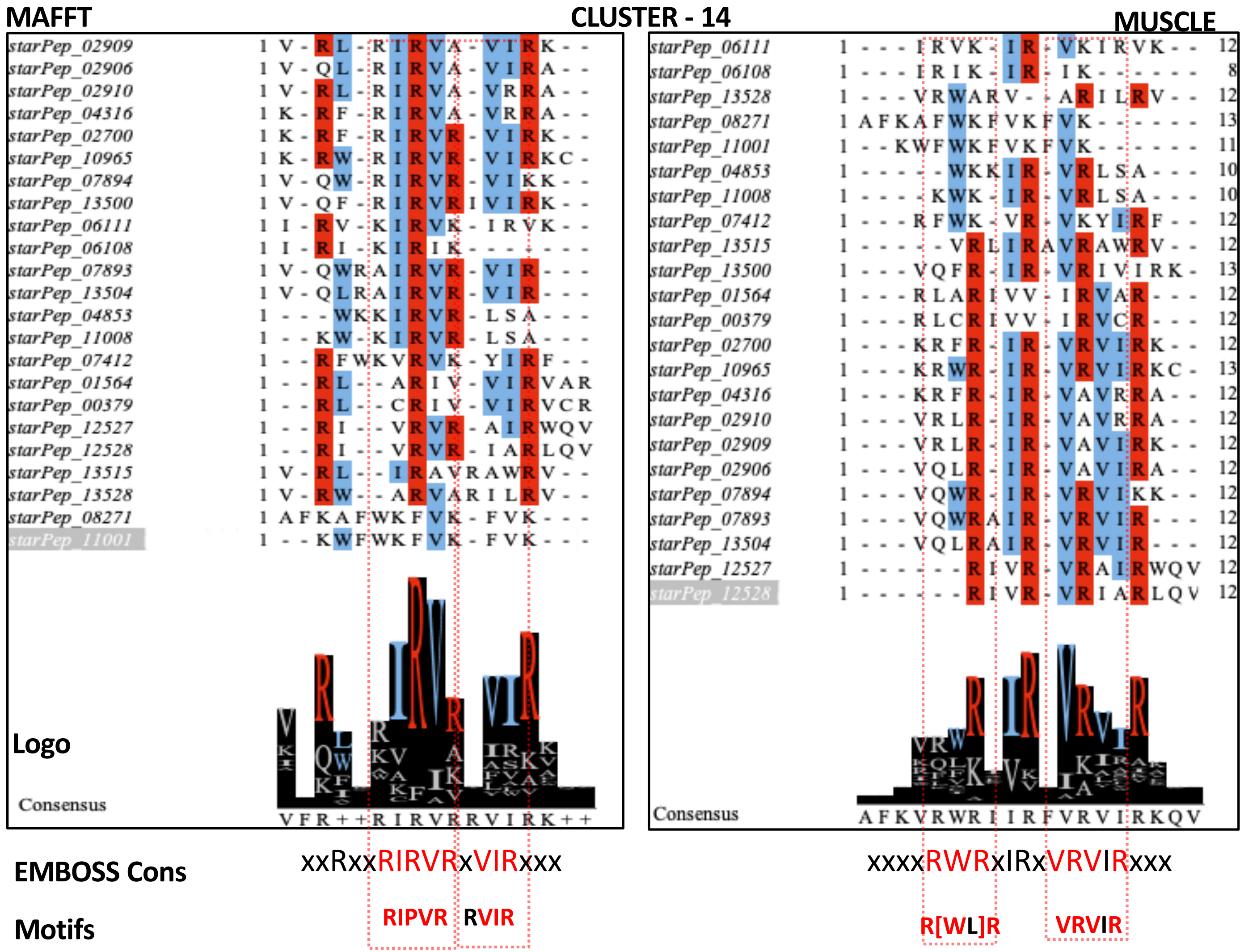
MAFFT
CLUSTER - 14
MUSCLE
Logo
Consensus
VFR++RIRVRRVIRK++
Consensus
AFKVRWRIIRFVRVIRKQV
EMBOSS Cons
xxRxxRIRVRxVIRxxx
xxxxRWRxIRxVRVIRxxx
Motifs
RIPVR
RVIR
R[WL]R
VRVIR

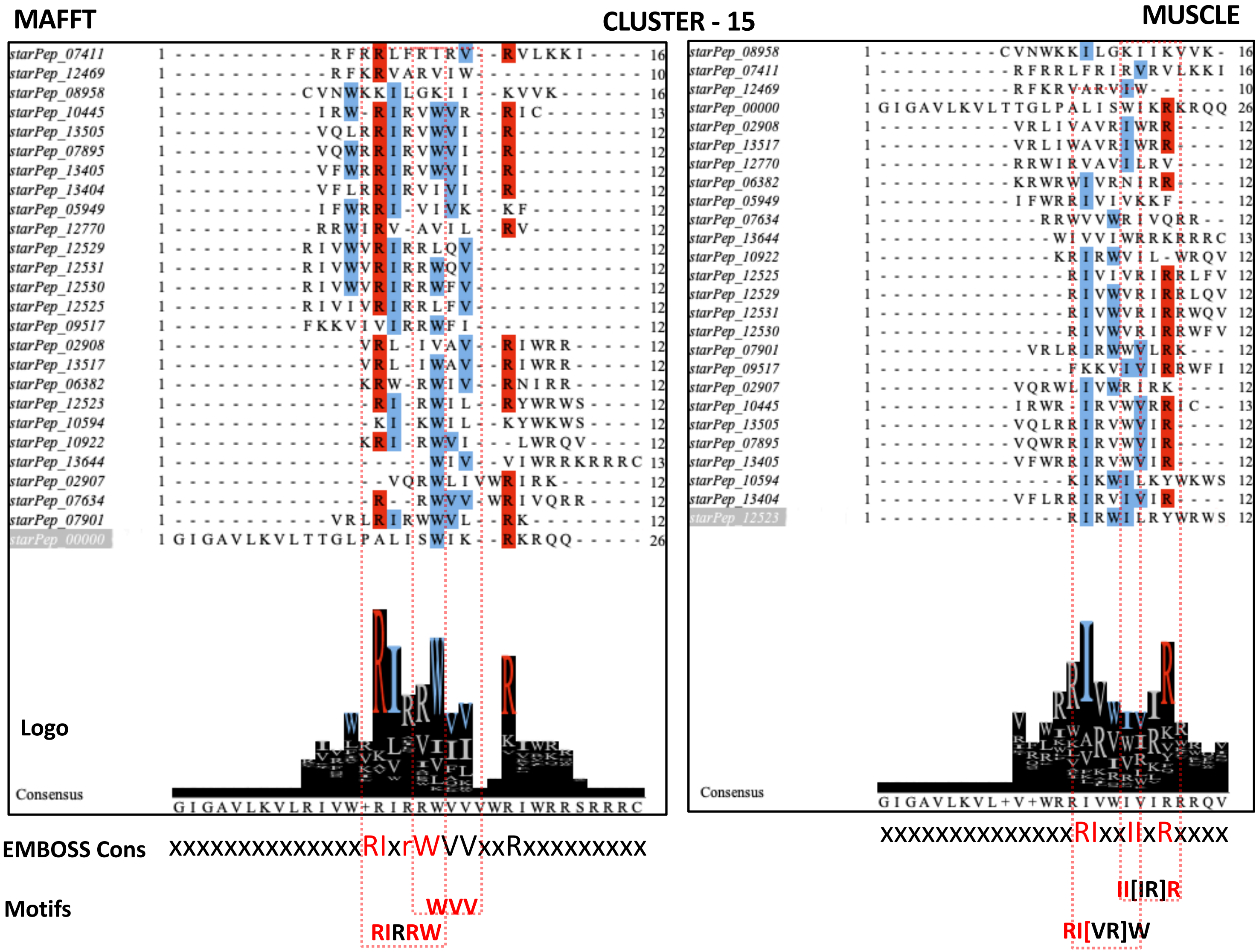
MAFFT
CLUSTER - 15
MUSCLE
Logo
Consensus
GIGAVLKVLRIVW+RIRRWVVVWRIWRRSRRRC
Consensus
GIGAVLKVL+V+WRRIVWIVIRRQV
EMBOSS Cons
xxxxxxxxxxxxxxRIxrWVVxxRxxxxxxxxx
xxxxxxxxxxxxxxRIxxIIxRxxxx
Motifs
WVV
RIRRW
II[IR]R
RI[VR]W

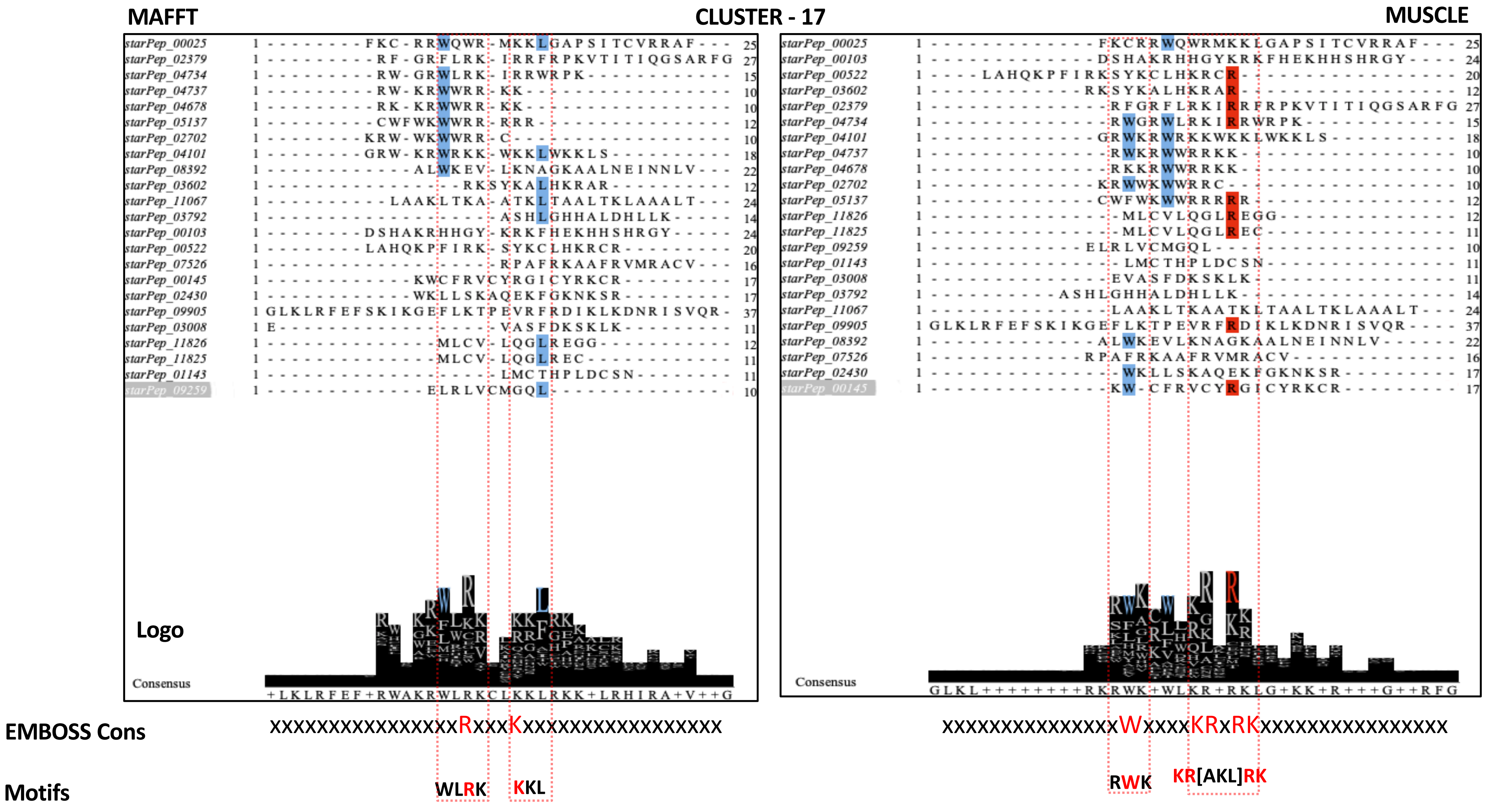
MAFFT
CLUSTER - 17
MUSCLE
Logo
Consensus
+LKLRFEF+RWAKRWLRKCLKKLRKK+LRHIRA+V++G
GLKL++++++++RKRWK+WLKR+RKLG+KK+R+++G++RFG
EMBOSS Cons
xxxxxxxxxxxxxxxxRxxxKxxxxxxxxxxxxxxxxx
xxxxxxxxxxxxxxWxxxxKRxRKxxxxxxxxxxxxxxxx
Motifs
WLRK
KKL
RWK
KR[AKL]RK

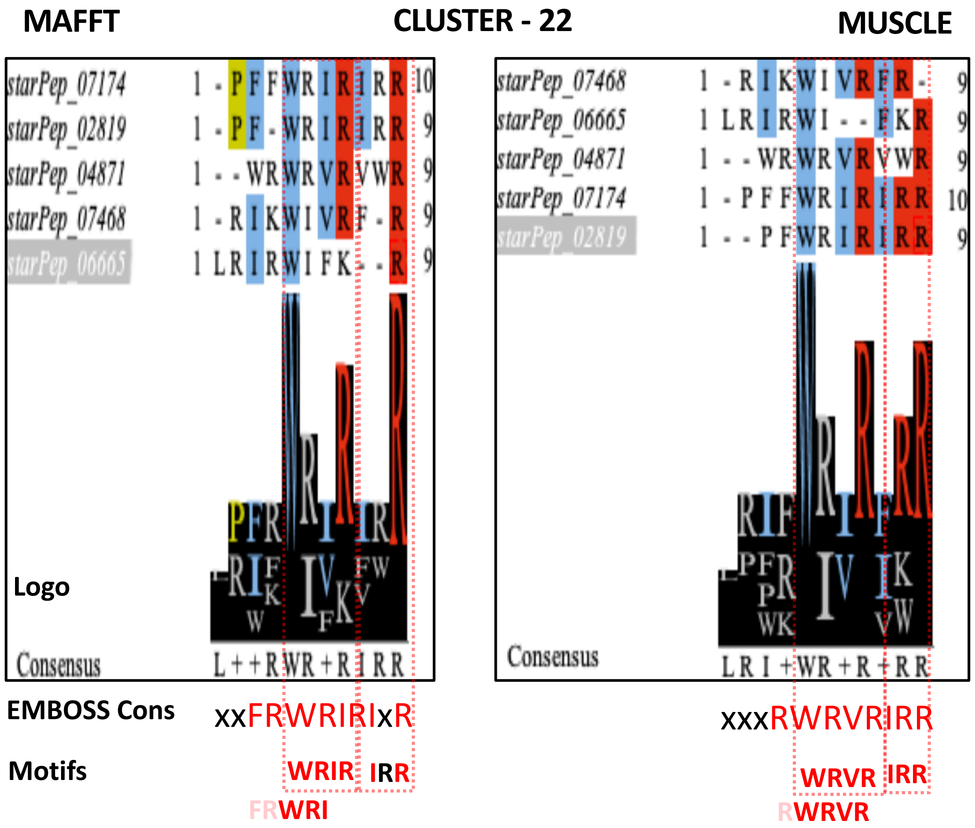

MAFFT
CLUSTER - 22
MUSCLE
starPep_07174
starPep_02819
starPep_04871
starPep_07468
starPep_06665
Logo
Consensus
L++RWR+RIRR
EMBOSS Cons
xxFRWRIRIxR
Motifs
WRIR
IRR
FRWRI
starPep_07468
starPep_06665
starPep_04871
starPep_07174
starPep_02819
Consensus
LRI+WR+R+RR
xxxRWRVRIRR
WRVR
IRR
RWRVR

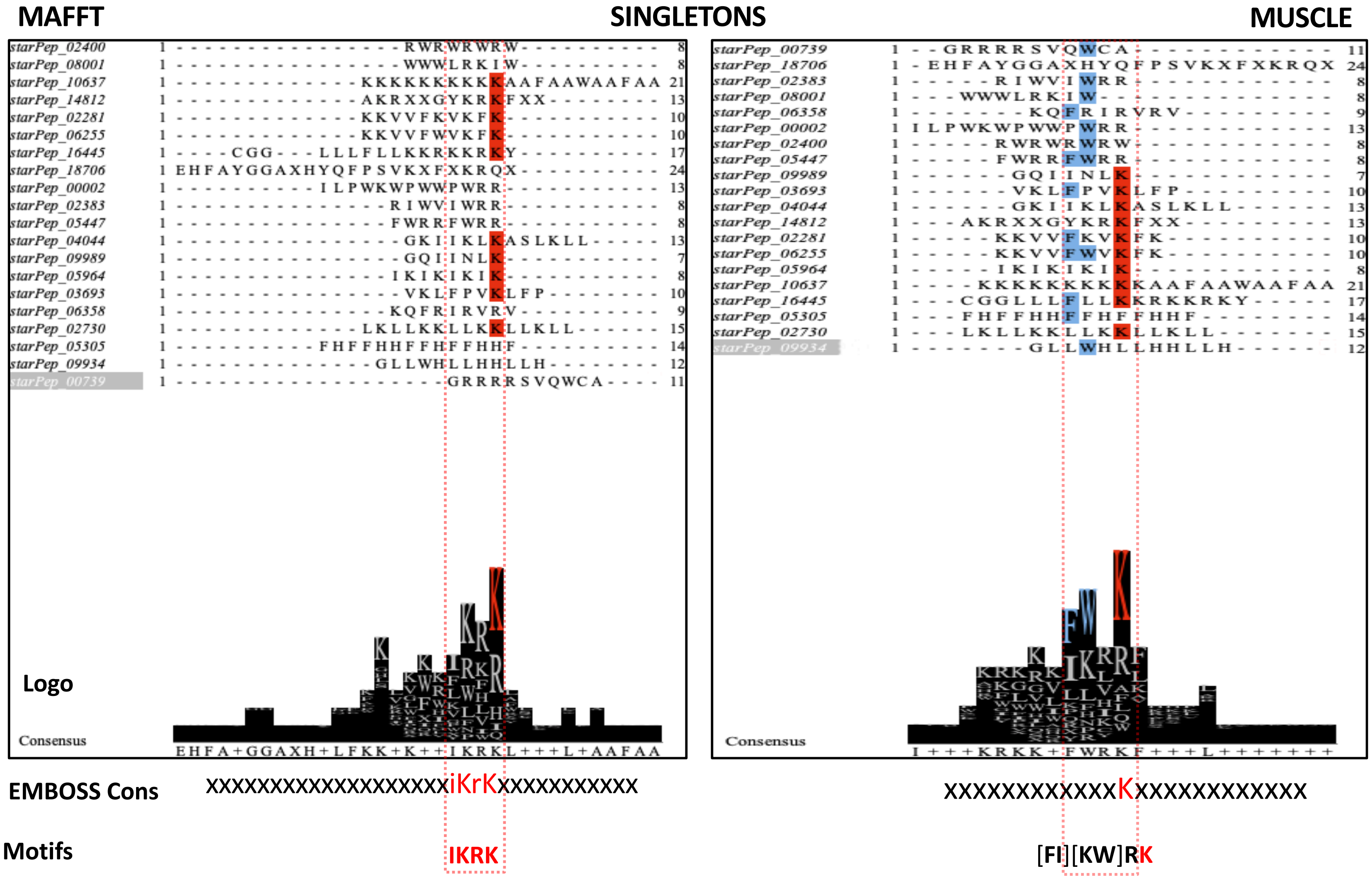
MAFFT
SINGLETONS
MUSCLE
Logo
Consensus
Consensus
EMBOSS Cons
XXXXXXXXXXXXXXXXXXXiKrKXXXXXXXXXXX
XXXXXXXXXXXKXXXXXXXXXXXX
Motifs
IKRK
[FI][KW]RK

	Local Alignment	Global Alignment
**Cluster 6**	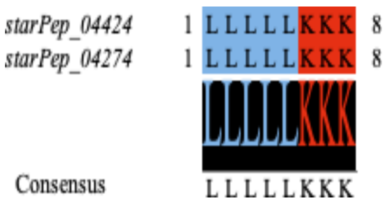	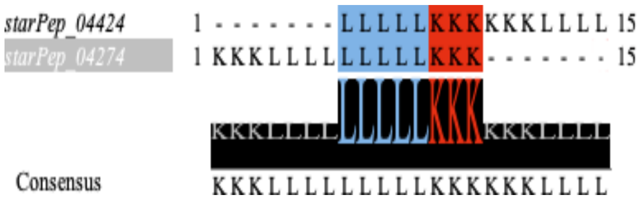
**Motifs**	LLLLLKKK	LLLLLKKK
**Cluster 28**	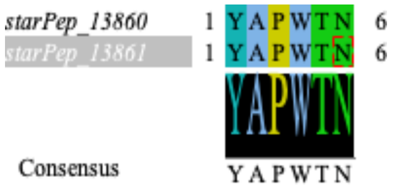	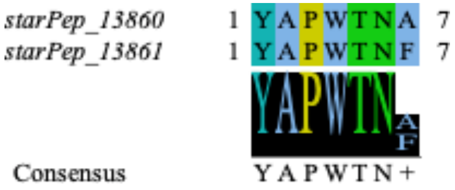
**Motifs**	YAPWYN	YAPWYN
