# Supplementary figures and images for: Complex Networks Analyses of Antibiofilm Peptides: An Emerging Tool for Next-Generation Antimicrobials’ Discovery
Source: Antibiotics (Basel). 2023 Apr 13;12(4):747. doi: 10.3390/antibiotics12040747 (PMC10135022; doi:10.3390/antibiotics12040747)

**Cutoff=0.35**

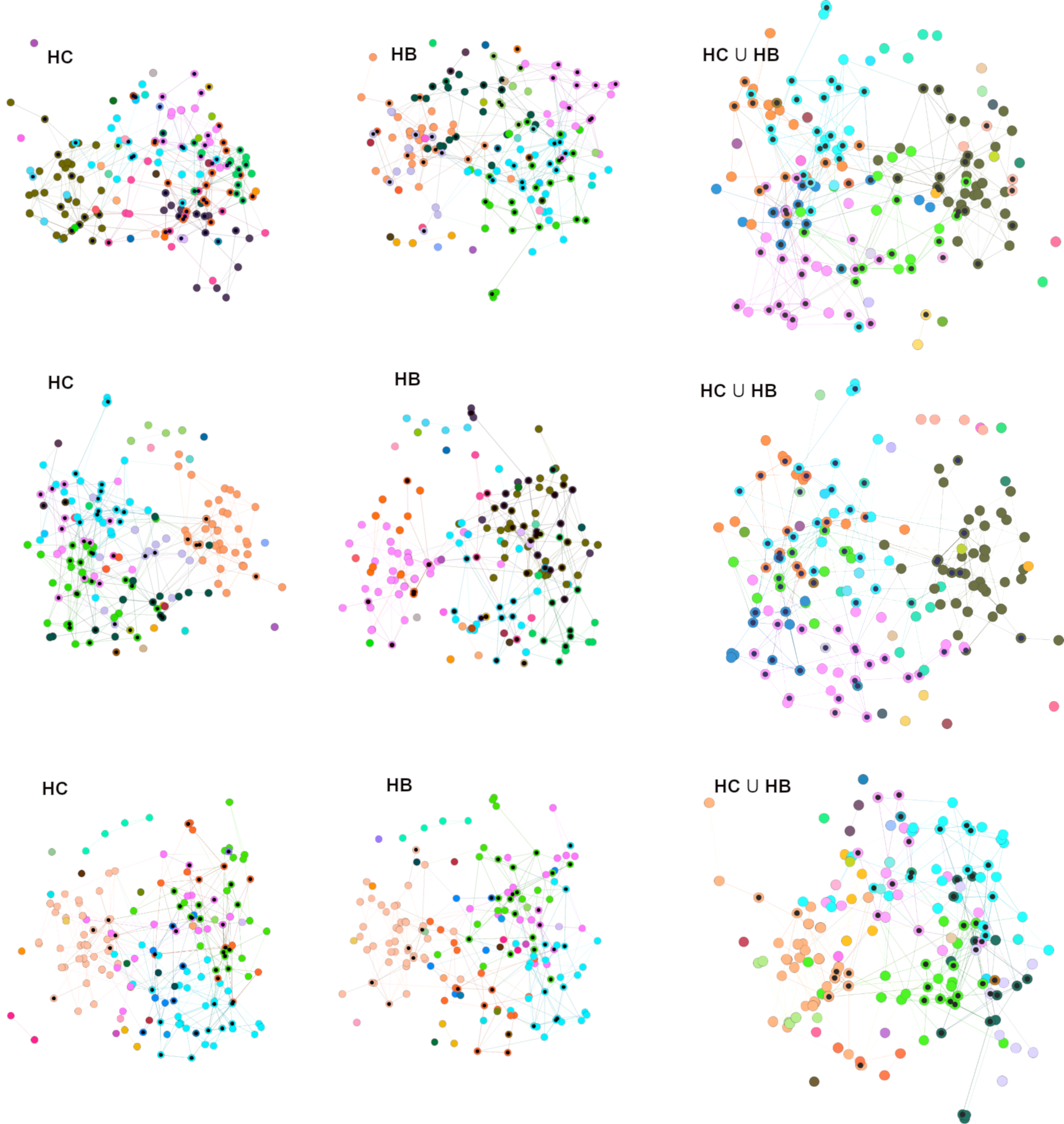

Supplement: Supplementary file 1 [file antibiotics-12-00747-s001.zip › Supplementary_Materials_Final/FigureS1_Overlapping_Subsets_6_7_8_HC_HB_&_HC-U-HB_for_Selection.pdf]

Cluster 4

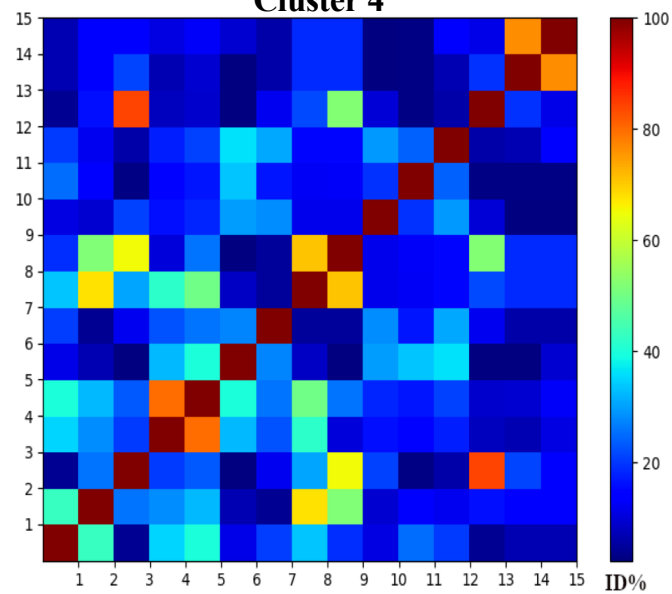

Cluster 7

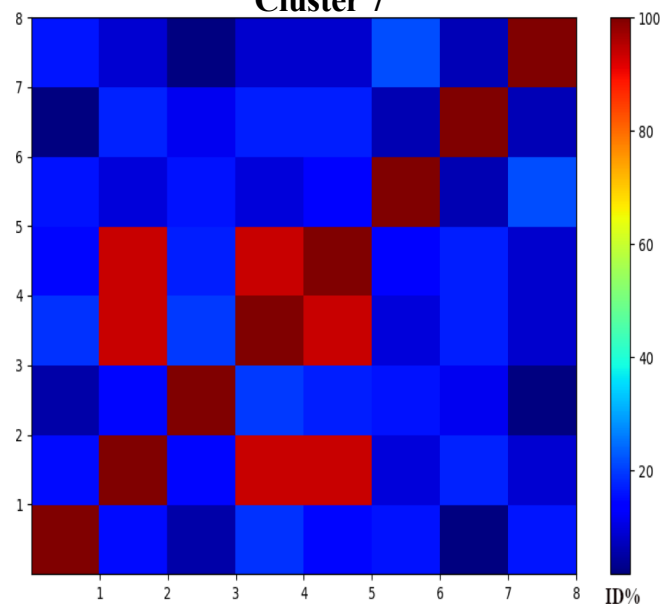

Cluster 9

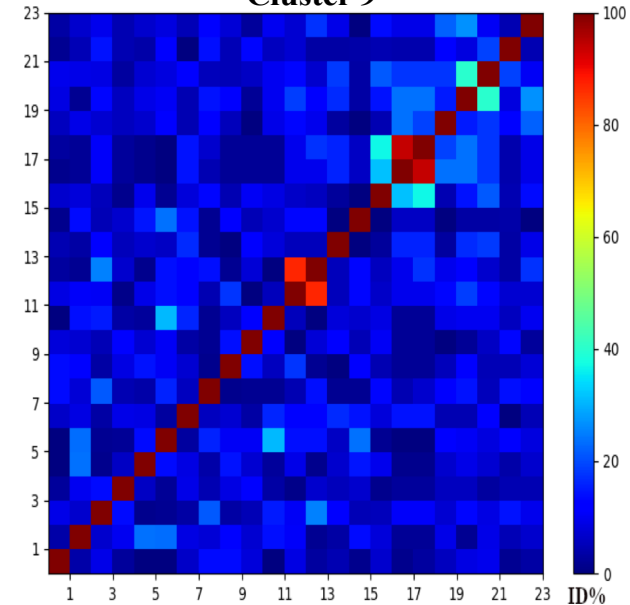

Cluster 11

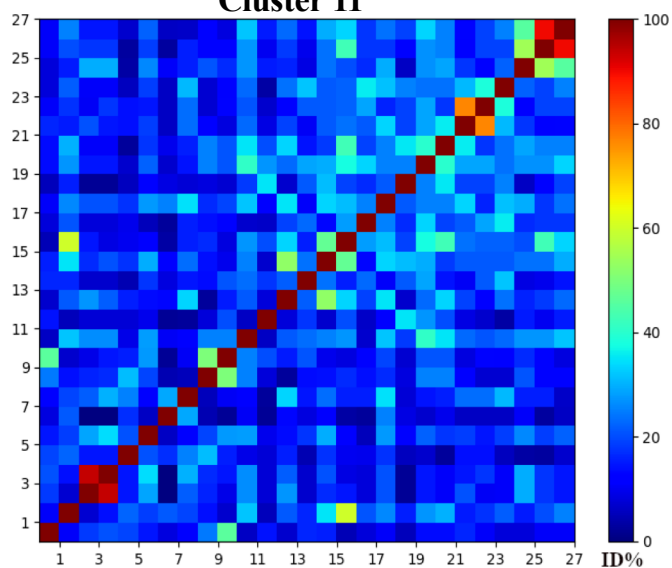

Cluster 14

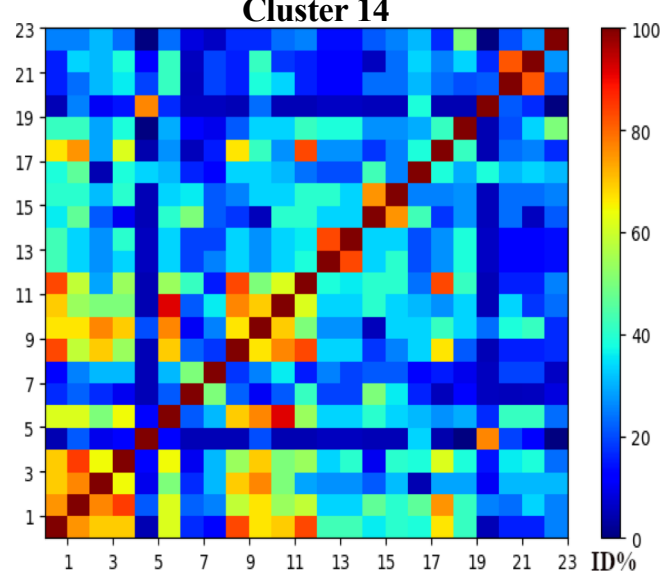

Cluster 15

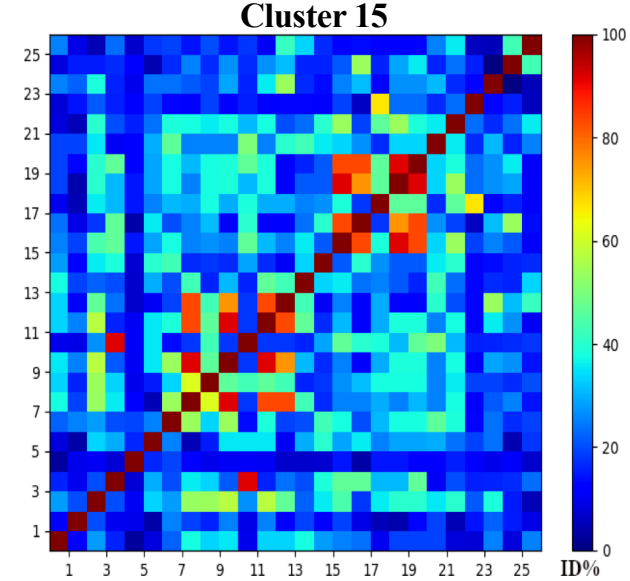

Cluster 17

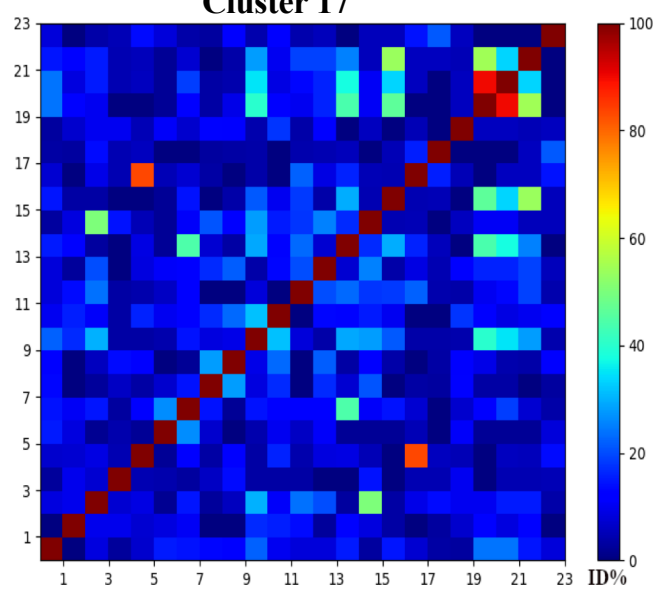

Cluster 22

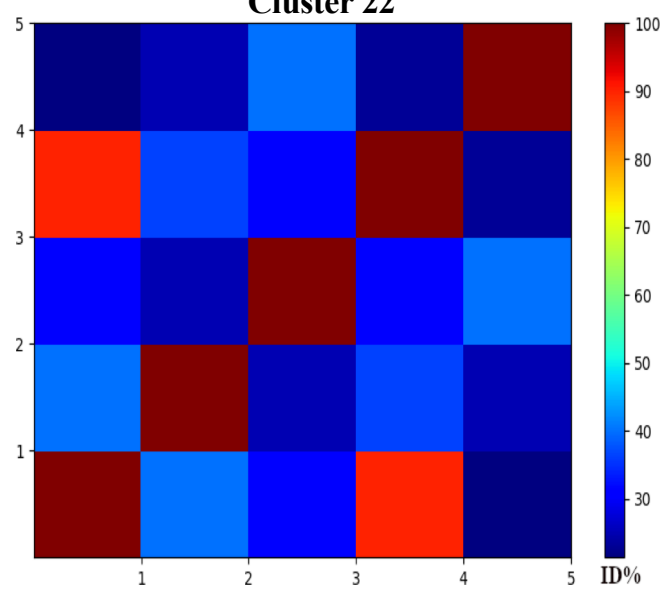

Singletons

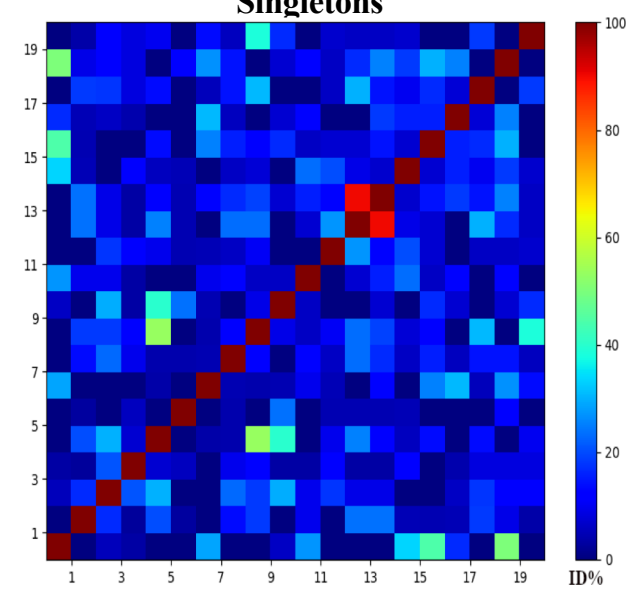

Supplement: Supplementary file 1 [file antibiotics-12-00747-s001.zip › Supplementary_Materials_Final/FigureS2_Heat_Maps_All_vs_All_Pairwise_Global_Identities_by_Commununity.pdf]
